# Microbial Ecology of Sulfur Mustard Toxicity: From Dysbiosis to Restoration

**DOI:** 10.3390/microorganisms13122793

**Published:** 2025-12-08

**Authors:** Xinkang Zhang, Guanchao Mao, Zhipeng Pei, Yunrui Sun, Jinfeng Cen, Shanshan Zhang, Songling Li, Wenqi Meng, Kai Xiao, Qingqiang Xu, Mingxue Sun

**Affiliations:** Lab of Toxicology and Pharmacology, Faculty of Naval Medicine, Naval Medical University, Shanghai 200433, China

**Keywords:** sulfur mustard, gut microbiota, dysbiosis, fecal microbiota transplantation

## Abstract

Sulfur mustard (SM) causes multi-organ toxicity, yet its impact on intestinal tissue and the associated gut microbiota remains poorly characterized. This study demonstrates that in a mouse model of SM exposure, gut microbial ecological collapse occurs, characterized by depletion of protective taxa (Bifidobacteriales, *Gordonibacter*, and Lachnospiraceae UCG010) while promoting a 302-fold expansion of inflammation-associated *Escherichia/Shigella*. Mendelian randomization analysis established causal relationships between these SM-perturbed taxa and human inflammatory bowel disease. Fecal microbiota transplantation effectively restored microbial diversity (Simpson index: 0.85 to 0.95), suppressed *Escherichia/Shigella* by 97.4%, and ameliorated intestinal pathology. Longitudinal tracking revealed persistent vulnerability of Bifidobacteriales compared to other depleted taxa. Our findings establish the gut microbiota as a key mediator in SM intestinal toxicity and provide new insights for microbiota-targeted interventions against chemical injuries.

## 1. Introduction

Sulfur mustard (SM), a potent chemical warfare agent, poses significant threats to human health due to its ability to cause severe multi-organ toxicity [1,2,3,4,5]. The gastrointestinal tract demonstrates particularly vulnerability to SM exposure, primarily attributable to its high epithelial turnover rate [6]. While the direct toxic mechanisms of SM through alkylation reactions are well established, including mucosal barrier disruption, epithelial necrosis, and inflammatory infiltration [7,8], the potential involvement of the gut microbiota in modulating SM toxicity remains largely unexplored. This knowledge gap is particularly noteworthy given accumulating evidence that environmental toxicants can propagate tissue injury through gut microbiota dysbiosis [9,10,11].

The clinical progression of SM poisoning further underscores the importance of understanding its impact on gut microbiota. Following initial acute exposure, many survivors develop persistent gastrointestinal disturbances characterized by chronic indigestion, abdominal distension, epigastric pain, and gastric hypochlorhydria [12]. These chronic symptoms, which significantly impact patients’ quality of life, share notable similarities with certain functional gastrointestinal disorders and inflammatory bowel disease (IBD) in their clinical presentation [13,14]. This observation led us to hypothesize that gut microbiota alterations might represent a common underlying mechanism connecting acute SM exposure with long-term gastrointestinal complications.

The potential mechanisms by which SM may induce gut microbiota dysbiosis are multifaceted. As the gut microbiota serves as the first line of contact for many chemical agents [15], it may participate in the biotransformation of SM, potentially converting it into a more toxic or less toxic form and influencing its distribution and excretion. Furthermore, SM-induced mucosal barrier disruption could lead to the loss of mucus-associated anaerobic bacteria, alteration of oxygen gradients, and creation of favorable conditions for facultative anaerobes such as *Escherichia/Shigella*. Drawing parallels from studies on other environmental toxicants like heavy metals and persistent organic pollutants [16,17], SM may similarly activate specific inflammatory signaling pathways and induce oxidative stress responses, which in turn could drive gut microbiota dysbiosis. This dysbiosis may further exacerbate intestinal inflammation and barrier damage through mechanisms such as reduction in beneficial short-chain fatty acid (SCFA)-producing bacteria and an increase in pathogenic bacteria capable of producing toxins or activating inflammatory cascades.

To comprehensively investigate this hypothesis, we designed an integrated research strategy that progresses from observational findings to causal inference and functional validation. Our investigation commenced with 16S rRNA sequencing to characterize the structural alterations in gut microbiota following SM exposure, identifying specific microbial taxa affected by this chemical insult. Building upon these findings, we employed Mendelian randomization (MR) analysis to evaluate whether the SM-perturbed microbial taxa demonstrate causal relevance to IBD, thereby bridging experimental toxicology with human disease pathogenesis.

To establish functional causality, we performed fecal microbiota transplantation (FMT) experiments to determine whether restoring a healthy microbial ecosystem could mitigate SM-induced pathological outcomes. Finally, we extended our investigation to long-term SM survivors to examine the persistent effects on gut microbiota and identify potential recovery patterns. This sequential approach, progressing from characterization to causal inference, functional validation, and long-term observation, provides a robust framework for elucidating the role of gut microbiota in SM toxicity.

Through this multidisciplinary strategy, our study aims to not only advance the understanding of SM toxic mechanisms but also establish a novel paradigm for translating discoveries from toxicological models into clinically relevant insights for managing chemical exposure victims.

## 2. Materials and Methods

### 2.1. Animal Models and SM Exposure

Thirty male ICR mice (8 weeks old) were randomly allocated to three experimental groups (n = 10 per group): (1) a control group receiving propylene glycol vehicle injections; (2) an SM-exposed group in which the mouse intoxication model was established through subcutaneous injection of SM solution into the dorsal skin at a dose of 30 mg/kg body weight, following the established protocol by Pei [18]; and (3) an FMT-treated group subjected to SM exposure followed by fecal microbiota transplantation. The SM was dissolved in propylene glycol to achieve the required concentration prior to administration. Control animals received an equal volume of propylene glycol vehicle via the same route. All mouse studies strictly complied with the United States National Research Council’s Guide for the Care and Use of Laboratory Animals and were approved by the Ethics Committee of Medical Research at the Naval Medical University, Shanghai, China (Approval No. 20180308007), ensuring full adherence to ethical standards governing humane treatment of experimental subjects.

### 2.2. 16S rRNA Gene Sequencing and Analysis

Fecal DNA was extracted using the QIAamp PowerFecal Pro DNA Kit (Qiagen, Hilden, Germany). The V3-V4 hypervariable regions of bacterial 16S rRNA genes were amplified with primers 341F/806R, followed by paired-end sequencing on the Illumina NovaSeq platform (Illumina, San Diego, CA, USA). Raw sequences were processed in QIIME (version 1.9.1) with the following workflow: quality filtering and denoising via DADA2 to generate amplicon sequence variants (ASVs), followed by taxonomic classification using the SILVA reference database at 99% similarity. Microbial diversity analyses included α-diversity and β-diversity, performed in QIIME. Differentially abundant taxa were identified using linear discriminant analysis effect size (LEfSe, v1.0) with an LDA score threshold > 2.0 and Kruskal–Wallis test (*p* < 0.05) [19].

### 2.3. MR Analysis

To establish causal links between gut microbiota with inflammatory bowel disease (IBD) and pneumonia, we implemented MR analysis adhering to core assumptions: genetic instruments strongly associate with 211 microbial taxa, are independent of confounders, and affect IBD and pneumonia solely via microbiota. Exposure data for microbial taxa derived from MiBioGen consortium (N = 18,340; 16S rRNA regions V4/V3-V4/V1-V2; mbQTL mapping with age/sex adjustment) [20], while IBD outcome data (GWAS ID:ieu-a-31; N = 34,652) were sourced from MRC IEU OpenGWAS (https://gwas.mrcieu.ac.uk/datasets/; accessed on 31 July 2025). Instrumental variables underwent sequential filtering: genome-wide significant SNPs (*p* < 1 × 10^−5^), LD clumping (r^2^ < 0.001, 1 Mb window), palindromic SNP removal, and exclusion of weak IVs (F-statistic < 10; F = [R^2^/(1 − R^2^)] × [(N − K − 1)/K] where R^2^ = 2 × EAF × (1 − EAF) × β^2^). Primary causal inference used inverse-variance weighted (IVW) regression, supplemented by MR-Egger, weighted median, simple mode, and weighted mode methods. Sensitivity analyses included Cochran’s Q test for heterogeneity (*p* < 0.05 significant), MR-Egger intercept and MR-PRESSO tests for pleiotropy, and leave-one-out analysis for outlier detection.

### 2.4. Fecal Microbiota Transplantation

Fresh fecal samples from healthy donor mice were homogenized in sterile phosphate-buffered saline (PBS) at a concentration of 100 mg/mL. The homogenate was filtered through a 200-mesh sterile sieve (pore size ~75 μm) to remove large particulate debris while preserving bacteria and soluble components. The filtrate was then centrifuged at 500× *g* for 5 min (4 °C) to pellet any remaining particulate matter. The resulting supernatant containing the microbiota was mixed with glycerol-PBS (10% *v*/*v*) under anaerobic conditions and stored at −80 °C until use. Recipient mice received daily oral gavage of 200 μL freshly thawed fecal suspension for 7 consecutive days using stainless steel feeding needles. Fecal samples for microbial analysis were collected one week after the completion of the gavage protocol. Successful microbial engraftment was verified via 16S rRNA gene sequencing comparing pre- vs. post-FMT microbiota profiles, with >30% donor strain retention defined as effective colonization [21].

### 2.5. Intestinal Histopathological Analysis

Intestinal tissue samples were collected from all experimental groups following the completion of interventions. Tissue specimens were fixed in 4% paraformaldehyde for 24 h, processed through graded ethanol series, embedded in paraffin, and sectioned at 5 μm thickness. Sections were stained with hematoxylin and eosin (H&E) following standard protocols. Histopathological evaluation was performed by two independent pathologists blinded to the experimental groups, focusing on epithelial integrity, inflammatory cell infiltration, glandular architecture, and mucosal damage severity. Representative images were captured using a light microscope. Histopathological scoring was conducted based on the extent of epithelial damage, inflammatory cell infiltration, and structural alterations, with consistent findings observed across multiple tissue sections from each group.

### 2.6. Statistical Analysis

All data are presented as the mean ± standard deviation. Statistical analyses were conducted using IBM SPSS Statistics v26.0. Group comparisons were performed with ANOVA followed by LSD post hoc tests for parametric data. All statistical assumptions, including normality (Shapiro–Wilk test) and homogeneity of variance (Levene’s test), were verified prior to analysis.

## 3. Results

### 3.1. SM Exposure Alters Gut Microbiota Composition and Diversity

**Figure 1:**
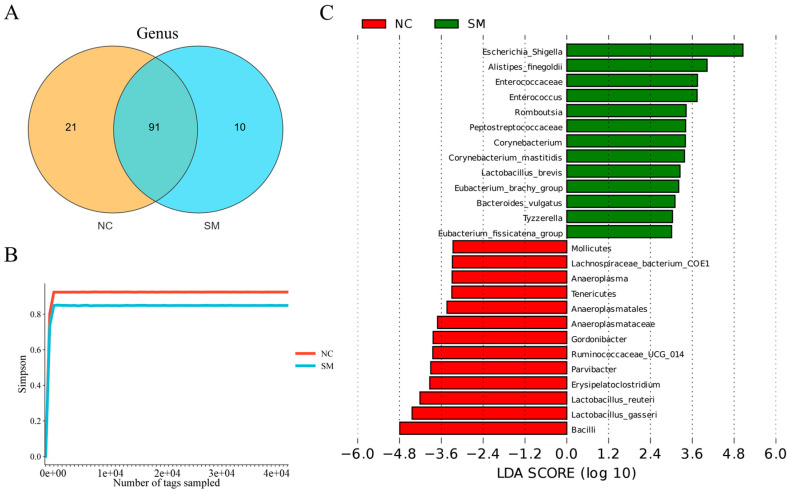
Sulfur mustard exposure alters gut microbiota composition and diversity. (**A**) Distribution of unique and shared bacterial OTUs between normal control (NC) and sulfur mustard-exposed (SM) groups (Venn diagram). (**B**) Comparative α-diversity assessed by Simpson index (ANOVA-LSD). (**C**). Taxonomic distribution of signature microbiota at genus level (LEfSe analysis, LDA > 3.0).

16S rRNA sequencing analysis revealed that SM exposure induced substantial restructuring of the gut microbiota. Although 91 bacterial operational taxonomic units (OTUs) were shared between the control and SM-exposed groups (Figure 1A), distinct SM-associated dysbiosis was observed, marked by the exclusive presence of 10 OTUs in exposed subjects (e.g., ASF356, *Glutamicibacter*, *Asticcacaulis*), compared to 21 unique OTUs in controls. Concurrently, a marked reduction in α-diversity was detected in the SM group, with Simpson indices decreasing from 0.92 to 0.85 (Figure 1B). LEfSe analysis further identified *Escherichia/Shigella* as the most differentially enriched taxon in exposed specimens (Figure 1C), suggesting selective expansion of this inflammation-associated pathobiont [22]. Together, these findings indicate that SM exposure triggers a collapse of the gut microbial community, characterized by diminished diversity and enrichment of pathogenic taxa.

### 3.2. Mendelian Randomization Analysis of Gut Microbiota–IBD Associations

**Figure 2:**
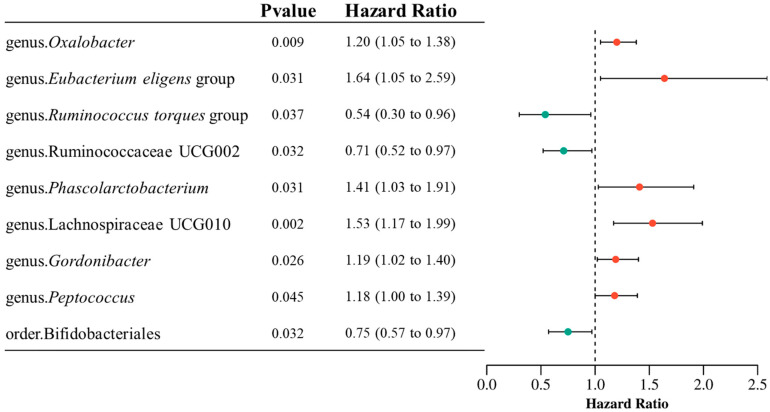
Forest plots showing the causal effects of 9 gut microbiota taxa on IBD from MR analysis.

To investigate the potential causal relationship between the gut microbiota and IBD, we performed a MR analysis to investigate genetically predicted gut microbiota–IBD associations. After rigorous instrumental variable selection, 784 SNPs were included in the final analysis. Using the inverse variance weighted (IVW) method, we identified nine bacterial taxa that were significantly associated with IBD risk. Three taxa exhibited protective effects: *Ruminococcus torques* group (genus level; OR = 0.54, 95% CI: 0.30–0.96, *p* = 0.037), Ruminococcaceae UCG002 (genus; OR = 0.58, 95% CI: 0.35–0.95, *p* = 0.032), and Bifidobacteriales (order; OR = 0.75, 95% CI: 0.57–0.97, *p* = 0.032). Conversely, six taxa were associated with an increased risk of IBD: *Oxalobacter* (genus; OR = 1.21, 95% CI: 1.00–1.46, *p* = 0.009), *Eubacterium eligens* group (genus; OR = 1.64, 95% CI: 1.05–2.59, *p* = 0.031), *Phascolarctobacterium* (genus; OR = 1.41, 95% CI: 1.03–1.91, *p* = 0.031), Lachnospiraceae UCG010 (genus; OR = 1.43, 95% CI: 1.01–2.04, *p* = 0.002), *Gordonibacter* (genus; OR = 1.19, 95% CI: 1.01–1.40, *p* = 0.026), and *Peptococcus* (genus; OR = 1.18, 95% CI: 1.00–1.39, *p* = 0.045). No SNPs were excluded following PhenoScanner V2 screening. The full set of associations is presented in Figure 2.

### 3.3. Concordance Analysis of MR-Derived Taxa with 16S Sequencing Data

**Figure 3:**
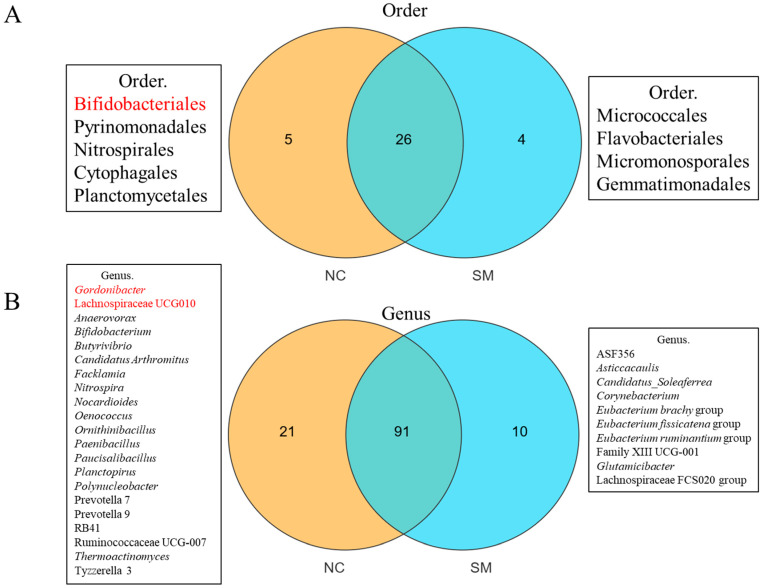
Venn diagrams of gut microbiota taxa with MR-validated IBD associations. (**A**) Order-level and (**B**) genus-level Venn diagrams comparing control and SM-exposed groups, highlighting taxa validated by Mendelian randomization analysis as causally associated with inflammatory bowel disease.

By integrating MR findings with 16S rRNA sequencing data, we identified several IBD-associated microbial taxa that were depleted following SM exposure. At the order level, Bifidobacteriales, previously identified as a protective taxon against IBD, was detected exclusively in the control (NC) group and was completely absent in SM-exposed subjects (Figure 3A). At the genus level, *Gordonibacter* and Lachnospiraceae UCG010, both identified as risk-enhancing taxa for IBD in the MR analysis, were also found to be eliminated after SM exposure (Figure 3B). The convergence of these experimental and genetic epidemiology approaches provides supportive evidence that SM exposure disrupts specific gut microbiota that are genetically implicated in IBD. The loss of both protective and risk-modulating taxa suggests potential mechanisms through which SM may influence IBD pathogenesis by affecting key bacterial groups involved in maintaining intestinal homeostasis.

### 3.4. FMT Ameliorates SM-Induced Intestinal Injury and Restores Microbial Homeostasis

**Figure 4:**
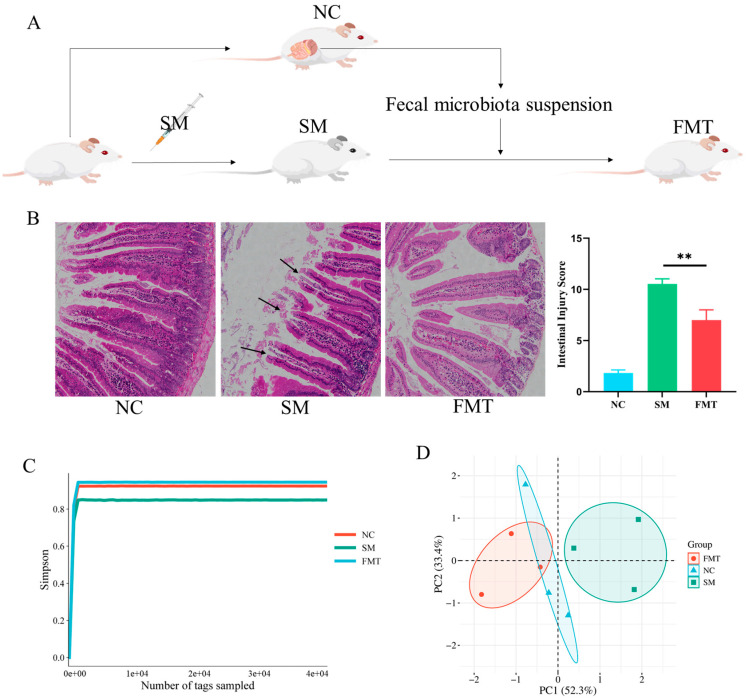
Microbial diversity dynamics following experimental interventions. (**A**) Diagram of the experimental protocol; (**B**) representative histopathological sections of intestinal tissues. Comparison of pathological intestinal injury scores in SM-exposed mice. ** *p* < 0.01; (**C**) Simpson index comparison across groups (ANOVA-LSD); (**D**) NMDS ordination based on Bray–Curtis dissimilarity.

Fecal microbiota transplantation from healthy donors effectively reversed SM-induced intestinal damage and gut microbial dysbiosis. The experimental workflow illustrating the FMT procedure is presented in Figure 4A. Histopathological examination revealed distinct morphological changes among the experimental groups (Figure 4B). The normal control group exhibited intact tissue architecture with regularly arranged glandular epithelial cells, clear cellular stratification, and minimal inflammatory cell infiltration, demonstrating preserved mucosal integrity. In contrast, the SM-exposed group showed severe structural disruption characterized by epithelial sloughing, disordered cellular arrangement, and significant inflammatory cell infiltration, indicating substantial mucosal injury. Notably, the FMT-treated group demonstrated considerable improvement, with restored structural integrity, more regular cellular organization, and reduced inflammatory infiltration compared to the SM-exposed group.

At the ecosystem level, α-diversity analysis showed that the significantly reduced Simpson index in SM-exposed mice (0.85) compared to controls (0.92) was restored to 0.95 in FMT-treated mice (Figure 4C), indicating not only recovery but potential overcompensation of microbial richness. Analysis of β-diversity using NMDS based on Bray–Curtis distances revealed marked shifts in microbial community structure (Figure 4D). While SM exposure induced a clear separation from the control cluster, FMT recipients clustered closely with the control group and were distinctly separated from the SM-exposed animals. These findings collectively demonstrate that FMT effectively re-establishes both mucosal integrity and a healthy-like microbial ecosystem following SM-induced disruption.

### 3.5. FMT Mediated Restoration of Gut Microbiota Composition

**Figure 5:**
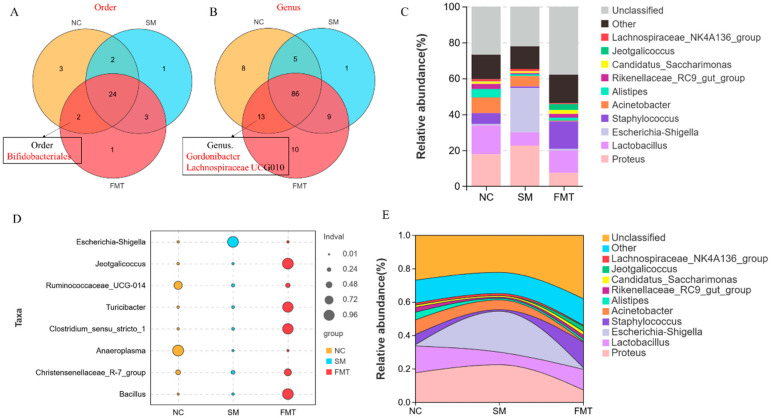
FMT restores gut microbial composition and structure. (**A**) Order-level and (**B**) genus-level Venn diagrams comparing taxa distribution among control, SM-exposed, and FMT-treated groups. (**C**) Relative abundance of major bacterial taxa across experimental groups. (**D**) Differential abundance of indicator species. (**E**) Stacked area plot displaying compositional changes of microbial taxa of microbial community restructuring.

Analysis of the post-FMT microbial profiles revealed a broad restoration of gut microbiota composition. Critically, the three IBD-associated taxa previously depleted by SM exposure, Bifidobacteriales, *Gordonibacter*, and Lachnospiraceae UCG010, were successfully re-established in the transplanted mice, confirming the efficacy of FMT in modulating gut microbial structure. Concomitantly, FMT intervention induced a significant reduction in the abundance of *Escherichia/Shigella*. This genus had exhibited a dramatic 302-fold increase in SM-exposed mice, reaching 15,283 reads compared to merely 498 in controls. Following transplantation, its abundance was reduced to 526 reads, representing a 97.4% decrease that brought it back to near-baseline levels. Other taxa, including *Lactobacillus* and *Proteus*, also exhibited significant shifts in abundance across experimental groups, though to a lesser extent than *Escherichia/Shigella*. Together, these results demonstrate that FMT facilitates a multi-faceted restructuring of the gut microbiota, characterized by the recovery of depleted beneficial taxa and the suppression of potential pathobionts such as *Escherichia/Shigella*, which may serve as an indicator of pathogenic shift in SM-induced dysbiosis (Figure 5).

### 3.6. Longitudinal Analysis of Microbial Dynamics in SM-Exposed Survivors

**Figure 6:**
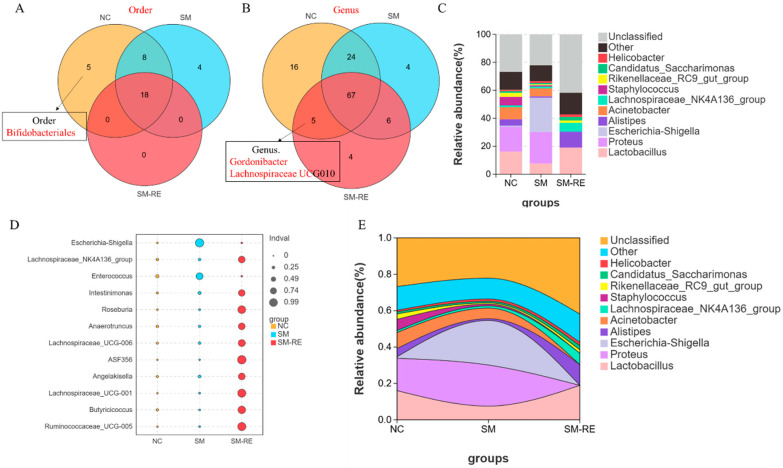
Gut microbiota dynamics during long-term recovery after SM exposure. (**A**) Order-level and (**B**) genus-level Venn diagrams comparing taxa distribution among control, SM-exposed, and long-term recovery (SM-RE) groups; (**C**) relative abundance of major bacterial taxa across experimental groups; (**D**) differential abundance of indicator species; (**E**) stacked area plot displaying compositional changes of microbial taxa throughout the recovery process.

To further investigate the resilience and recovery patterns of gut microbiota following SM exposure, we conducted a longitudinal analysis of surviving mice after four weeks of monitored recovery. Comparative analysis between these survivors and healthy controls revealed distinct patterns of microbial restitution among the previously depleted IBD-associated taxa. Notably, Bifidobacteriales at the order level failed to re-establish in the survivor population, indicating persistent depletion of this protective taxon. In contrast, both *Gordonibacter* and Lachnospiraceae UCG010 at the genus level demonstrated successful recolonization in the surviving mice, suggesting differential recovery capacities among the affected bacterial groups (Figure 6).

Concurrent analysis of pathogenic indicators showed that the abundance of *Escherichia/Shigella*, which had been dramatically elevated during acute SM exposure, returned to levels comparable to those in healthy controls following the recovery period. This normalization of the previously expanded pathobiont coincided with the overall clinical improvement observed in the surviving animals. These findings reveal heterogeneous recovery patterns within the gut ecosystem following SM-induced dysbiosis, with implications for understanding the long-term microbial consequences of chemical exposure and the inherent restorative capacity of the gut microbiota.

## 4. Discussion

This study provides a comprehensive investigation of gut microbiota dynamics in SM toxicity through an integrated approach combining 16S sequencing, MR, FMT, and longitudinal observation. Our findings reveal a distinct pattern of microbial dysbiosis characterized by selective depletion of specific IBD-associated taxa and dramatic expansion of pro-inflammatory pathobionts, establishing gut microbiota as both a biomarker and mediator in SM-induced intestinal pathology.

The specific microbial signature observed in this study, characterized by the disappearance of protective Bifidobacteriales and risk-associated *Gordonibacter* and Lachnospiraceae UCG010, coupled with a 302-fold expansion of *Escherichia/Shigella*, represents a unique response to SM exposure that differs from other chemical injuries [23,24]. This pattern aligns with emerging concepts in microbial ecology suggesting that chemical stressors can drive microbial communities into alternative stable states through selective pressure on specific taxa [25,26]. The complete absence of Bifidobacteriales in SM-exposed subjects, contrasted with its consistent presence in controls, underscores its potential role as a key protective taxon vulnerable to SM toxicity. These findings gain additional significance when considered alongside studies demonstrating the anti-inflammatory properties of Bifidobacteriales through its enhancement of gut barrier function and modulation of regulatory T cells [27,28].

Building upon these observations, our exploration of potential mechanisms, while necessarily speculative given the limited direct research on SM-gut microbiota interactions, suggests several plausible pathways. Drawing from established findings on other environmental toxicants, SM may disrupt gut microbiota through multiple potential mechanisms: (1) direct alteration of microbial composition through activation of specific signaling pathways (e.g., LPS/TLR4) [29]; (2) disruption of mucus-associated anaerobes and subsequent inflammatory cascades [30]; (3) interference with microbial metabolic functions, particularly short-chain fatty acid production [31]; and (4) alteration of receptor-mediated signaling pathways critical for maintaining intestinal homeostasis [32]. These mechanistic hypotheses, though requiring further validation, provide a valuable framework for understanding how SM-induced epithelial damage may lead to the observed microbial dysbiosis.

Our MR analysis provided valuable insights for bridging observations from animal models to human disease contexts [33]. The identification of SM-perturbed taxa that also show genetic associations with IBD in humans adds biological plausibility to our experimental findings [34]. Notably, the limited overlap between the broader set of MR-identified IBD-associated taxa and those actual responsive to SM exposure highlights the specificity of SM’s effect on gut microbiota. This specificity may help explain the distinct clinical presentation of SM-induced gastrointestinal damage compared to spontaneous IBD. Our integrated approach strengthens the interpretative framework of the study by combining genetic evidence with experimental observations, while appropriately acknowledging the conceptual rather than directly causal nature of this cross-species connection [35].

The differential recovery patterns observed in long-term survivors provide valuable insights into microbial resilience and ecological succession following chemical injury. The natural recolonization of *Gordonibacter* and Lachnospiraceae UCG010, contrasted with the persistent absence of Bifidobacteriales, suggests varying adaptive capacities among microbial taxa. This differential resilience may underlie the chronic gastrointestinal symptoms observed in SM survivors and echoes findings from other studies of microbial community recovery after environmental perturbations [36,37]. The failure of Bifidobacteriales to recover naturally highlights the potential need for targeted interventions to restore this functionally important taxon.

The dramatic expansion of *Escherichia/Shigella* and its effective suppression through FMT position this pathobiont as a central player in SM pathology. As an established pro-inflammatory genus with documented roles in intestinal barrier disruption [22], its 302-fold increase provides a plausible mechanism for SM-induced mucosal damage. The 97.4% reduction in *Escherichia/Shigella* abundance following FMT, coupled with distinct improvements in intestinal histopathology including restoration of epithelial integrity and reduction in inflammatory cell infiltration, demonstrates the therapeutic potential of targeting this pathobiont. This finding resonates with studies in other inflammatory conditions where *Escherichia/Shigella* expansion correlates with disease severity and responds to microbial-directed therapies [38].

The success of FMT in simultaneously restoring depleted beneficial taxa and suppressing pathobiont overgrowth demonstrates the potential of ecosystem-level interventions for chemical injuries [39,40]. The restoration of microbial diversity to levels exceeding baseline, paralleled by the recovery of intestinal mucosal structure, suggests that FMT can not only reverse SM-induced damage but potentially enhance ecological resilience. This aligns with the concept of “ecological therapy” aimed at restoring microbial community structure and function rather than targeting individual pathogens [41]. The concomitant improvement in both microbial profiles and histopathological features following FMT provides compelling evidence that microbial changes are active drivers rather than passive consequences of tissue damage, highlighting the crucial role of microbiota reconstruction in repairing SM-induced intestinal injury.

Several limitations warrant consideration in interpreting our findings. First, while our murine model effectively recapitulates key aspects of SM-induced dysbiosis, validation in human SM victims is essential to account for interspecies differences in microbiota composition and host response. Second, the precise mechanisms through which SM exposure selectively targets specific bacterial taxa remain to be fully elucidated. Potential mechanisms could include differential sensitivity to alkylating agents, variation in DNA repair capabilities, or competition for ecological niches under stress conditions. Third, the sample size of 10 mice per group, though consistent with established protocols, and the exclusive use of male mice represent limitations that future studies with larger cohorts including both sexes could address. Finally, the relatively small overlap between MR-identified IBD-associated taxa and SM-responsive microbes highlight the context-dependent nature of microbial pathogenesis and suggests that SM toxicity operates through both shared and unique pathways compared to spontaneous inflammatory conditions.

Our findings suggest several promising directions for both basic research and clinical translation. The identification of specific microbial signatures associated with SM injury provides potential diagnostic biomarkers for exposure assessment and severity stratification. The success of FMT supports further development of microbiota-targeted therapies, possibly including defined consortia of the depleted beneficial taxa or targeted approaches against *Escherichia/Shigella*. Future studies should also explore the molecular mechanisms underlying taxon-specific vulnerability to SM and the ecological principles governing community recovery, potentially informing more effective intervention strategies.

In conclusion, our multi-layered investigation establishes gut microbiota as a critical mediator of SM toxicity and identifies specific, reproducible changes in microbial ecology that drive pathological progression. By integrating findings from animal models with human genetic data and interventional studies, we provide a comprehensive framework for understanding and treating chemical-induced intestinal injury through microbiota-targeted approaches. The distinct pattern of microbial dysbiosis characterized by selective taxon depletion and pathobiont expansion, combined with the demonstrated efficacy of FMT, advances our understanding of environmental toxicant effects on microbial ecosystems and offers new avenues for managing chemical exposure victims.

## 5. Conclusions

This study demonstrates that sulfur mustard exposure induces gut microbiota dysbiosis through selective depletion of protective taxa (e.g., Bifidobacteriales) and promotes expansion of *Escherichia/Shigella*. Mendelian randomization established causal links between these perturbed microbiota and inflammatory bowel disease. Fecal microbiota transplantation effectively restored microbial ecology and alleviated intestinal injury, while longitudinal analysis revealed differential recovery patterns among bacterial taxa. These findings establish gut microbiota as a key mediator in SM toxicity and provide new insights for microbiota-targeted interventions against chemical injuries.

## Data Availability

The original contributions presented in this study are included in the article. Further inquiries can be directed at the corresponding authors.

## Abbreviations

The following abbreviations are used in this manuscript:

SM	Sulfur mustard
IBD	inflammatory bowel disease
MR	Mendelian randomization
FMT	Fecal microbiota transplantation
